# Publishing with purpose: Bringing lived experiences into journal development

**DOI:** 10.1371/journal.pmen.0000635

**Published:** 2026-06-11

**Authors:** Karli Montague-Cardoso, Parth Sharma, Bethan Burnside, Akiko Inoshima, Carlos A. Larrauri, Minh Dung Hoang Le, Taylor Locke, Victor Ugo, Luiz Roberto Carvalho, Charlene Sunkel

**Affiliations:** 1 PLOS: Public Library of Science, Cambridge, United Kingdom; 2 iHEAR, Sangath, Bhopal, India; 3 Independent; 4 The University of Tokyo, Tokyo, Japan; 5 Harvard TH Chan School of Public Health Executive and Continuing Education Program, Boston, Massachusetts, United States of America; 6 aves Mental Health (Global Mental Health Peer Network), Paarl, Western Cape, South Africa; 7 School of Psychology, The University of Sydney, Camperdown, New South Wales, Australia; 8 Mentally Aware Nigeria Initiative, Lagos, Nigeria; 9 MHPSS Collaborative, Copenhagen, Denmark; 10 SoulBeeGood, São Paulo, Brazil; 11 Vertentes Ecossistema de Saúde Mental, São Paulo, Brazil; 12 National Alliance on Mental Illness NAMI-NYS, New York, United States of America; 13 University of São Paulo, São Paulo, Brazil; PLOS: Public Library of Science, UNITED KINGDOM OF GREAT BRITAIN AND NORTHERN IRELAND

Since its inception, *PLOS Mental Health* has been driven by the overarching mission of bringing lived experience perspectives into the mental health publishing landscape in a way that is meaningful, unwavering, and inclusive of all communities and identities [1]. As a scholarly journal, we have a responsibility to maintain a multifaceted and authentic relationship with our communities, ensuring that we are not just accessible to them but also shaped by them. This requires a careful, flexible approach that is never executed as an afterthought and is not restricted to published content only.

For too long, people with lived experiences of mental health struggles have been excluded or have been included in a retrospective and tokenistic fashion. We have tried to remain accountable to our mission and have fully embraced this journey in which we are continuously improving. Alongside our published content [2–5], we have launched a number of intentional blog and seminar series that provide avenues for perspectives that might otherwise be excluded. However, despite such initiatives being carefully considered and well received by our readers, if we continue to give communities what we think they need or want without consultation, it can inadvertently make us complicit in exclusion and thus suppression.

Therefore, in 2024, we opened a call for applications to our Lived Experience Advisory Board (LEAB). It was crucial to us that this board included people with lived experience from across the globe, who were independent of the journal and could steer us toward what communities need, rather than just giving us retrospective feedback. The response to the call was overwhelmingly positive, and we formed our inaugural group of 13 members, some of whom are joining us as guest authors on this editorial. When we initially launched the call, there were understandable concerns regarding the lack of remuneration. It was therefore our responsibility not only to ensure that the inclusion of this group was meaningful, but also that the members felt valued, empowered, and enriched by their involvement with the journal. We do however still acknowledge that the lack of financial remuneration remains a significant barrier and a point of tension in lived experience engagement. Within the current constraints of our existing publishing model, we have focused on providing non-monetary value that challenges traditional academic hierarchies. This includes providing a platform for first-author publications - often otherwise gatekept by institutional affiliations - direct mentorship in the peer-review process, and the ability to steer the journal’s strategic direction, which we discuss in more detail below. We view this as a transitional phase while we work toward more sustainable and equitable models of compensation for expertise.

In this Editorial, we will be discussing what we have learned so far from this initiative and the people involved. In particular, we will outline what we believe are crucial aspects of integrating meaningful lived experience into scholarly publishing.

## Transparency and clarity

An initiative that serves as a tick box or is performative will be recognised as such very quickly. Trust is fragile, especially when it comes to something as sensitive as lived experience. Initiatives that aim to bring a lived experience perspective into publishing, or any industry, must be committed to delivering on carefully designed aims, which the advisory board is not only informed of but can also help shape. When the LEAB was set up for *PLOS Mental Health*, the journal shared clear, documented terms of reference with the group so they could read the information at their own pace and understand exactly what being a group member entailed and what they could expect from the journal. Within this documentation and the first meeting, it was explained to the members of the group that the journal was seeking to not only receive feedback on what had been done so far, but to understand more about topics that were important to the community and to be in a more informed position to make content more accessible.

In order to accommodate the different ways in which LEAB group members preferred to prepare for meetings, an agenda and detailed notes were shared prior to the first meeting so that those who wished to familiarise themselves with the material beforehand, could do so. This, however, was completely optional as all information was presented in the first meeting. Group members were then given space to contribute to the discussion. The group chair also communicated their ‘open door’ policy via email so that members could provide comments more discreetly and at a later date, if preferred. In the interest of online safety and confidentiality, no meetings were recorded, and no AI note takers were enabled. All notes were handwritten by the meeting chair and referred only to action points for the journal, rather than to any information about individuals within the group. Relational safety is integral to meaningful lived experience engagement; however, it risks remaining an abstract concept without tangible efforts to integrate it. By actively taking these steps (i.e., offering the choice whether to opt in for disclosure of lived experience, no mandatory camera presence, listening-in options, pre-shared agendas, and follow-up notes), relational safety was centred and reflected in the interactions during and beyond the meetings.

## Cultivating authentic connections

Anyone who chooses to participate in a lived experience focus group demonstrates a high level of trust and vulnerability. They are not required to share exactly what their experience is, but by openly identifying as someone who has lived experience of a mental health condition or struggle, they are sharing something about themselves that many might understandably never share with others, let alone a group of strangers, due to ongoing stigmatisation and discrimination. Time needs to be taken to build trust and authentic relationships. Members of lived experience groups need to see that those leading the initiative recognise the privilege they have been given by group members and the importance of a relationally safe environment. Not only are those with lived experience of mental health struggles showing trust by identifying as they do and sharing their expertise, but they have usually been harmed or suppressed in the past by organisations and systems that can have a very similar dynamic to the current group that they are part of.

Whilst there is an eagerness to rush through introductions and get to the actionable items of a meeting, the *PLOS Mental Health* LEAB took time to foster connections, not just between group members and the journal, but between the group members themselves. This is important because the members of the group will also be strangers to each other, and sharing opinions in such spaces requires developing trust. In short, group members were invited to introduce themselves using information that they were comfortable sharing. The group chair also shared their own experiences. As a journal, we feel it is important for our Editors to do this when comfortable, as they are demonstrating a level of trust in others that they hope to receive in return. Genuine trust is two-way. However, we are clear that the depth of personal disclosure remains entirely at the discretion of the individual. We do not view the sharing of trauma as a “price of entry” for participation, nor do we measure the value of a member’s expertise by the perceived severity or narrative arc of their personal history. The goal of this openness is to level the power dynamic, not to mandate vulnerability.

## Accountability and attribution

If a LEAB is set up but the organisation or people that the LEAB is advising does not transparently communicate the objectives of the group, does not deliver, and does not report to the group as they would to colleagues, then experts by experience are simply not being treated as experts. Without this accountability, the LEAB is no more than a tick box.

Everyone has their limits in both professional and personal settings. They may be limited in resources, knowledge, or capacity. This is not something that all focus group or advisory board members will automatically understand unless there is full transparency, because the restrictions in place can be industry-specific and are often norm-based. This does not, however, mean that limitations can be an excuse for taking no action. At *PLOS Mental Health*, we believe there is always something that can be done that is a step in the right direction.

Understandably, many will delay actions or avoid them altogether if they feel that they cannot do things ‘properly’ and completely deliver on something. In some cases, this hesitancy is necessary and responsible. However, we believe that not everything can and should be one big gesture. Most efforts are small, intentional, and most importantly, consistent. By making small changes here and there (in addition to some larger ones), we are laying the foundations for a transformative change. We are demonstrating that we can move publishing in a direction that is no longer exclusionary or insular. The world is far bigger than academic institutes, and advances require more than academic scholars alone.

In line with this philosophy, since the *PLOS Mental Health* LEAB was first formed last year, the journal has been making small changes that, crucially, are publicly attributed to this group and its members. Attribution is extremely important because, again, unless you attribute changes to the experts who suggested or inspired them, you are not treating them as experts. You are, in fact, guilty of subterfuge.

Based on suggestions from group members, we have been making changes to improve our accessibility and community engagement. We launched a blog series featuring press release highlights, and the journal’s Executive Editor started a regular LinkedIn newsletter. These gave the journal an opportunity to explore content in a more accessible, context-rich manner. We also created workshops and seminars for early career researchers and advocates who may not have had extensive experience with academic writing. As the LEAB pointed out, if we wished to provide a platform for lived experience, including peer reviewed, published content, then we needed to provide guidance for its potential authors that would otherwise be reserved only for those in academia. While initiatives like a LinkedIn newsletter or a blog series might appear as minor editorial outputs, we view them as critical low-barrier entry points designed to bypass the traditional academic paywall of complex jargon and formal peer-review hierarchies. These platforms allow us to disseminate insights in real-time and in formats that are accessible to the communities most affected by mental health research, rather than restricting knowledge to those with institutional credentials. By prioritising these small changes, we are intentionally eroding the insular nature of scholarly publishing and creating a more porous boundary between academic evidence and lived reality.

In addition, many of our blogs and seminars over the course of the year focused on topics specifically identified by our LEAB, including LGBTQ+ mental health, refugee inclusion, and structural injustices. We are also pleased to share that we published two Opinions and an Essay from Parth Sharma, one of the members of this group, which focus on the dangers of disinformation [6], lived experience in ethics committees [7], and a call for reparations, with a powerful critique of the mental health industrial complex [8]. Being able, as a journal, to publish work from our LEAB is a very proud moment. By shifting the journal’s power structure, we moved away from a model where experts by training speak about communities, to one where the LEAB dictates the discourse. The enrichment for members has been tangible: they have transitioned from advisors to lead authors on published content [6–8], gained expertise in scholarly publishing and peer-review ethics, and successfully challenged the journal to abandon sanitised academic formats in favour of more authentic, embodied knowledge.

Finally, behind the scenes, we also convened an Academic Editor focus group to learn more about regional variations in open science practices [9], research culture, and ethics. This group is directly inspired by comments from the LEAB regarding the considerable variations across and within the regions in which they reside.

## Reflecting and evolving

Importantly, we do not see a LEAB as a destination or a result. We see it as the *start* of a journey. Not only has the journal changed as a result of the expertise offered by its LEAB, but the board’s role has also evolved based on its feedback. The very name of the group, for instance, has changed. When we first launched the group, it was called the Lived Experience Focus Group. This has, however, been changed to the LEAB based on feedback to reflect the group’s essential contributions and signal our commitment to meaningfully embedding that expertise in decision-making. Furthermore, publishing work from this group and writing an Editorial such as this one were not among the group’s initial objectives. These have evolved purely as a result of the group members expressing their interests, capacities, and priorities. As we move forward, we will continue to evolve the LEAB not only to ensure that we are changing as a journal but also to ensure that group members continue to feel valued.

We wanted to do more than have members of our LEAB as co-authors. We wanted to give them a voice. As such, we would now like to share individual statements from members of the LEAB that reflect on their experiences with the journal.

*Parth Sharma:*

*‘Volunteering is a political act informed by the ability of someone to remain activated enough to engage. As a person with multiple marginalized identities, who chooses to politicize their lived experience, there is almost an underlying assumption that we must (and can) volunteer our time to institutions that have harmed us owing to a bland reformist agenda that serves as a proxy for equity. I was optimistically cautious when I first saw the call for advisors, owing to my relationship with academia. Academic Journals have a long history of legitimizing the epistemic authority of technical experts, researchers and practitioners on matters of mental health. Authority that has perpetuated existing inequities against people with lived experience and only recently changed with the shared global commitment by funding organisations, journals and researchers to engage in co-design. A commitment that in itself is evolving and recognising the need for various methods of meaningful collaboration.*

*It was from the very onset of the advisory group that I felt a sense of great comfort with the intentionality in engagement and reflexivity practiced by those with institutional power. The meetings were not pre-defined goals by PLOS Mental Health, that required contextualisation by the advisors. They were spaces for raw, unsanitized discussion and dissent on the field of mental health, our positionality within it and the actions we could move towards. It is rare to see systemic changes incorporated into organizations, and everything we discussed was reflected as either an accepted change or as a part of an ongoing process. Across the year long engagement, what truly made a difference was the joy of connecting with each other and the support provided by the editor and team at PLOS Mental Health to spotlight our voices. With the rising disinformation campaign around Autism, I got an opportunity to lead an opinion piece co-authored by twelve neurodivergent people from across the world, who chose to resist including LEAB member Taylor [6]. It was also during this time that PLOS Mental Health published the peer reviewed essay on Reparations that I wrote as a call-in to the ‘Lived Experience’ field in Global Mental Health [8]. An essay, written to defy structural formats of academia, initiated global discourse, because the editor, and the journal felt the need to champion it. In both these instances and ever since, I felt recognised and affirmed as an expert.*

*Publishers remain a crucial part of the lived experience ecosystem, and this engagement with PLOS Mental Health is evidence of the fact that authentic, transparent and meaningful collaboration is possible when the editors spearhead this change.’*

*Bethan Burnside:*

*‘What surprised me about being part of the inaugural PLOS Mental Health Lived Experience Advisory Board was the authentic intentionality from which it stemmed. From the very first meeting, it was clear to me that we weren’t being asked to validate the journal’s existing positions and endeavours but instead invited to unsettle them. The space has carved and held room for dissent, dreaming, and the messy, necessary work of (re)imagining mental health futures that center care, community-rooted knowledge, and disability justice. That kind of proactive, uncomfortable reflexivity isn’t common in my previous experience as a lived experience expert, where my identity and position have so often been co‑opted to legitimize organizational agendas, and treated as add‑ons to mental health scholarship. The editors didn’t just listen, but actually implemented structural changes and provided a platform for work that pushes against the grain on bridging divides between research and embodied knowledge, and on rebuilding trust where it has been broken between academia and the broader community. My involvement with PLOS Mental Health has reminded me just how much publishers — alongside other key actors, such as curators, cultural workers, grassroots organizers, and others straddling lines between science and society — can play a meaningful role in shifting power, redistributing authority, and making space for knowledge that emerges from care, community, and the lived realities of those most impacted by mental health systems. It has affirmed that these kinds of collaborations can foster a site of repair and possibility when done transparently, reflexively, and with genuine accountability.’*

*Akiko Inoshima:*

*‘Having been based in Japan while engaging across East Asia—including Korea and China—I have been shaped by cultural contexts that place great value on attentiveness to others, social harmony, and coexistence, often over individual assertion. This has, in many ways, enriched who I am. At the same time, however, I have found that it can also wear down something more fundamental, compounded by social stigma: the sense that I, as a person with lived experience, am allowed to speak—and even to feel and express anger. It is for this reason that being part of the LEAB has felt both meaningful and transformative. What stands out is not only the openness of the editors but the genuine effort to create a space that feels equal and psychologically safe. Just as importantly, there is a clear awareness that we each come from different cultural and national contexts, and that these differences matter in how we speak, listen, and engage.*

*Much of the human rights movement for persons with psychosocial disabilities since the 1980s and community-based mental health welfare, coined by Dr. Basaglia, were mainly led by Western philosophy-based mindsets. While there has certainly been exchange and mutual influence, international solidarity has often remained at the level of civil society—frequently connected through the internet, yet not always fully integrated across different domains. In particular, lived experience advocacy has largely been rooted in NGOs and grassroots spaces, and has only more recently begun to find a place within academia and more formal understandings of “expertise”. In this context, PLOS Mental Health feels both timely and important. It is not only creating space for lived experience within academia, but also asking a more complex question: how can people from different cultural contexts meaningfully work together in a world that is increasingly interconnected, yet uneven in power, voice, and representation?*

*As someone rooted in East Asia, I believe that ideas and practices become meaningful only when thoughtfully and thoroughly localized. Simultaneously, I firmly believe localization does not preclude exchange. In fact, it necessitates an openness to be shaped by others and to shape in return. Moving forward, I believe this balance will become even more important. This Editorial itself emerged from that kind of openness. When I first shared the idea of co-developing an editorial piece with Karli, it was received with warmth and immediacy, and quickly brought into form. That responsiveness—simple as it may seem—reflects a way of working that makes participation feel possible.*

*Looking ahead, as the 21st century continues to reshape our understanding of expertise—particularly in the context of AI and rapidly globalized knowledge systems—the question of how mental health communities and academia will adapt becomes ever more pressing. Initiatives such as this offer not only a response but a direction of travel.*

*Carlos A. Larrauri:*

*‘As an aspiring scholar, it is essential that I work within spaces where knowledge beyond empirically derived sources is respected and valued. Lived experience—direct, first-person encounter with mental health conditions and the expertise that emerges from it—offers something that conventional research methods alone cannot: insight into the subjective dimensions of illness, the gaps between clinical intention and patient reality, and the questions that most urgently need asking. Too often, however, the voices of those with lived experience have been marginalized or treated as secondary to empirical inquiry rather than recognized as a distinct and necessary complement to it.*

*If our goal is to improve the lives and care of people with mental health conditions, we cannot fully achieve it without centering their perspectives and treating them as co-equal contributors to knowledge production. This includes creating opportunities for people to make sense of their own experience through scholarship and advocacy—a process that is itself recuperative, because the act of transforming suffering into meaning and knowledge restores a sense of agency that illness so often disrupts. Such work not only facilitates personal development and recovery but may also improve the lives of others.’*

*Minh Dung Hoang Le:*

*‘Joining the PLOS Mental Health LEAB as a PhD scholar and clinical psychologist, and having navigated academic and clinical systems across North America, Asia, and now Australia, has underscored the necessity of decentering Western‑centric research hierarchies. My experience with the Board has moved beyond consultation to witnessing a meaningful shift in who is recognised as a legitimate source of knowledge. By integrating diverse lived experiences into the core of journal development, we can ensure that mental health science is not only methodologically rigorous but also socially accountable and culturally resonant. This represents a move away from a universalised psychology toward an approach that respects the specificities of migration, language, and local community wisdom.’*

*Taylor Locke:*

*‘I have held many paid and voluntary roles within academic and academic-adjacent spaces, but none have demonstrated as strong a commitment to equitable representation and inclusion as my experience serving on the PLOS Mental Health Lived Experience Advisory Board. Karli’s leadership, grounded in her own lived experience, is evident in her efforts not only to ensure the journal is guided by diverse, global perspectives, but also to recognize and credit our contributions when they lead to meaningful change. This level of acknowledgment is rare in lived experience advisory structures, regardless of whether they are remunerated.*

*I have also greatly appreciated the opportunities for professional development and growth provided through the journal, which are thoughtfully integrated into the advisory experience and, again, uncommon in comparable roles. While the lack of remuneration is openly acknowledged, the journal’s transparent and intentional efforts to make this experience equitable and mutually beneficial despite these limitations are quite apparent. It sends a clear message that fair compensation is not solely about payment, but about ensuring lived experience voices are genuinely valued, respected, and uplifted.*

*As my dear friend and colleague Parth Sharma has said, “volunteering is a political act,” and I remain deeply grateful for the opportunity to use my privilege to support such an institution that exemplifies these ideals of meaningful lived experience inclusion.*

*Victor Ugo:*

*‘What has felt most important to me about this initiative, and about being part of the inaugural PLOS Mental Health Lived Experience Advisory Board, is that it engages lived experience at the level where journals help shape the field itself. Publishing plays a significant role in determining which questions are given space, which perspectives are treated as credible, and how knowledge travels into wider conversations across research, policy, and practice. In that sense, involving people with lived experience in journal development carries real value because it brings a broader and more grounded sense of relevance into editorial thinking. It helps draw attention to issues that might otherwise remain peripheral, it deepens judgment about what matters, and it strengthens the connection between scholarly work and the realities it is ultimately trying to speak to. It also has implications for accessibility, since people with lived experience often see clearly where language, format, and process create unnecessary distance between published knowledge and the communities most affected by it. What I have appreciated in PLOS Mental Health’s approach is the seriousness with which this has been taken as part of the journal’s ongoing development, because that creates better conditions for publishing that is more attentive to context, more accountable in its orientation, and more closely aligned with the lived realities at the heart of mental health.’*

*Luiz Roberto Carvalho:*

*‘Being part of the Lived Experience Advisory Board (LEAB) at PLOS Mental Health has been a deeply meaningful experience for me. More than an honor, it reinforces the strategic and meaningful, ethical value of lived experience with far more honesty and consequence. For too long, we have heard the language of inclusion without seeing a real shift in who sets priorities, who produces knowledge, and who is seen as legitimate enough to shape decisions. Stigma does not only appear in overt prejudice. It also persists when people with lived experience are kept at the margins, invited to consult but rarely trusted to influence. That is why I see the work PLOS Mental Health is doing through the LEAB as so important.*

*What is being built there is not symbolic participation. There is a real commitment to transparency, clarity, trust, and meaningful listening. And that changes the nature of the space. It moves lived experience out of the realm of tokenism and begins to recognize it for what it truly is - an essential knowledge that the field needs if it is serious about becoming more just, more accessible, and more coherent with the values it so often claims to uphold.*

*For me, witnessing this progress brings both joy and responsibility. Expanding the presence of people with lived experience is not simply a matter of representation. It is a matter of transformation. This is how we begin to dismantle long-standing barriers of stigma and open more legitimate pathways for care, for the democratization of knowledge, and for a science that does not only speak about people, but also with them and from them.’*

## Conclusions

As a journal, we want to acknowledge our responsibility and the unique position that we are in to bring together people from across the globe with different skills and experiences. Research and clinical advances are communicated to the world through journals. If journals do not evolve to incorporate and respect experts by experience in the way they currently incorporate and respect experts by training, then a meaningful and permanent integration of lived experiences across the mental health field cannot be fully realised. We have intentionally included members of the *PLOS Mental Health* LEAB as co-authors on this Editorial because they are part of the journal just as much as our Academic Editors are. We hope to continue to learn from each other so that we can make a real difference where and when it is needed most. Our LEAB will continue to evolve and whilst there have been successful elements to its implementation, we will always remain open and flexible. Without this, we cannot stay true to our mission.
